# Frontiers in oxytocin science: from basic to practice

**DOI:** 10.3389/fnins.2013.00250

**Published:** 2013-12-23

**Authors:** Idan Shalev, Richard P. Ebstein

**Affiliations:** ^1^Department of Psychology and Neuroscience, Duke UniversityDurham, NC, USA; ^2^Department of Psychology, National University of SingaporeSingapore

**Keywords:** oxytocin, CD38, receptor, intranasal, receptors, social behavior, psychiatry

Oxytocin is a paramount social hormone in mammals and accumulating evidence also strengthens a leading role of this nonapeptide in human social behavior. From voles to men, oxytocin modulates an intriguing number of social behaviors that resonates across species. Behaviors modulated by oxytocin include the quality of pair bonding, parenting, social stress, in-group and out-group relationships, and social communications. Recent molecular genetic studies of the human oxytocin receptor (*OXTR*) gene have strengthened the evidence regarding the role of this nonapeptide in a range of normal and pathological human behaviors. Moreover, dysfunctions in the oxytocin neural pathways are likely contributing to deficits in social skills and communication in several psychiatric disorders. Indeed, a growing number of clinical studies are now testing the therapeutic effect of intranasal oxytocin administration in mental disorders- from autism to schizophrenia.

Our social behavior is extraordinarily complex and is mediated by multiple and overlapping systems. Thus, it remains a challenging task to understand the neurobiology and neuroendocrinology underlying this complexity. One of the important determinants of human social behavior is the oxytocin system, which has provided a uniquely promising avenue of research toward revealing the tapestry of these complex social phenotypes. This Research Topic provide a broad view of the current state of oxytocin science in humans and illustrate how pharmacological, genetic, and neuroimaging strategies can be successfully combined toward unraveling the mystery of how human social behavior is regulated.

Part A provides several review articles outlining salient facets of current oxytocin research. Lopatina et al. (2013) focus on translational approaches and show how rodent models elucidate the roles of the *OXTR* and *CD38* genes in social behaviors. This review generates new perspectives in clinical therapies for diseases characterized by deficits in social cognition. Kumsta et al. (2013) provide an overview of the functional importance of *OXTR* promoter region methylation and summarizes a group of epigenetic studies that reveal the role of *OXTR* methylation signatures in shaping behavioral phenotypes. Quintana et al. (2013) highlight a key role for autonomic cardiac control in social behavior and psychiatric disorders and suggest the intriguing idea that autonomic cardiac control may moderate the relationship between oxytocin and social behavior. MacDonald (2013) discusses three factors that can influence individual responses to intranasal administration of oxytocin; sex and hormonal status, genetic variation, and attachment history. Finally, MacDonald and Feifel (2013) discuss the promise of oxytocin-based therapeutics and identify 10 key questions that future oxytocin research should address to facilitate oxytocin use in clinical medicine and truly deliver this molecule's therapeutic potential.

Part B provides several research articles from basic methodology to the powerful strategy combining molecular genetics and neuroimaging, so-called imaging genomics. van IJzendoorn et al. (2012) address the important question of how long salivary oxytocin levels remain elevated following intranasal administration and further examine the effect of different doses of oxytocin administration. Kret and De Dreu (2013) explore the effect of intranasal oxytocin in the context of group formation and further consider the effect of fetal testosterone and empathic concern on this process. Fischer-Shofty et al. (2013) test the effects of intranasal oxytocin administration on fear recognition in schizophrenic patients compared to healthy controls. Finally, Sauer et al. (2013) describe an imaging genomics approach using intranasal oxytocin administration during responses to social stimuli. Specifically, they investigate interactions between oxytocin and dopamine-related genes in contributing to individual differences in the activation of different brain regions.

Understanding social behavior in our species is not only clinically extremely relevant for disorders characterized by dysfunctional social relationships, but also research on human social hormones has important implications beyond the clinic. Revealing the biological roots of human social behavior impacts the Social Sciences, including economics, sociology and psychology and moreover, is of great interest to the public at large.

## Part A

1. Review Article
  The Roles of Oxytocin and CD38 in Social or Parental Behaviors
  Olga Lopatina, Alena Inzhutova, Alla B. Salmina and Haruhiro Higashida
2. Mini Review Article
  Epigenetic regulation of the oxytocin receptor gene: implications for behavioral neuroscience
  Robert Kumsta, Elisabeth Hummel, Frances S. Chen and Markus Heinrichs
3. Review Article
  A role for autonomic cardiac control in the effects of oxytocin on social behavior and psychiatric illness
  Daniel S. Quintana, Andrew H. Kemp, Gail A. Alvares and Adam J. Guastella
4. Mini Review Article
  Sex, Receptors, and Attachment: A Review of Individual Factors Influencing Response to Oxytocin
  Kai S. MacDonald
5. Review Article
  Helping oxytocin deliver: considerations in the development of oxytocin-based therapeutics for brain disorders
  K. MacDonald and D. Feifel

## Part B

1. Original Research Article
  Elevated Salivary Levels of Oxytocin Persist More than 7 h after Intranasal Administration
  Marinus H. van IJzendoorn, Ritu Bhandari, Rixt van der Veen, Karen M. Grewen and Marian J. Bakermans-Kranenburg
2. Original Research Article
  Oxytocin-Motivated Ally Selection is Moderated by Fetal Testosterone Exposure and Empathic Concern
  Mariska E. Kret and Carsten K. W. De Dreu
3. Original Research Article
  Characterization of the effects of oxytocin on fear recognition in patients with schizophrenia and in healthy controls
  Meytal Fischer-Shofty, Simone G. Shamay-Tsoory and Yechiel Levkovitz
4. Original Research Article
  Imaging oxytocin × dopamine interactions: an epistasis effect of CD38 and COMT gene variants influences the impact of oxytocin on amygdala activation to social stimuli
  Carina Sauer, Christian Montag, Martin Reuter and Peter Kirsch
